# Myocardial T_1_ mapping with spectrally-selective inversion pulse to reduce the influence of fat

**DOI:** 10.1186/1532-429X-18-S1-P19

**Published:** 2016-01-27

**Authors:** Maryam Nezafat, Sébastien Roujol, Jihye Jang, Tamer Basha, Rene M Botnar

**Affiliations:** 1 Division of Imaging Sciences & Biomedical Engineering, King's College London, London, UK; 2 Department of Medicine, Beth Israel Deaconess Medical Center and Harvard Medical School, Boston, MA USA; 3 Computer Aided Medical Procedures, Technische Universität München, Munich, Germany; 4 Biomedical Engineering Department, Cairo University, Giza, Egypt

## Background

Changes in the longitudinal relaxation time (T_1_) of the myocardium are considered an important imaging-based biomarker to detect and quantify diffuse fibrosis. Several sequences have been suggested to measure myocardial T_1_ values [1]. However, T_1_ measurements are often influenced by the presence of intramyocardial or epicardial fat [2,3,4]. The aim of this study was to minimize the effect of fat in T_1_ mapping by the use of a water selective inversion pulse and to investigate the impact of this spectrally-selective inversion pulse on T_1_ measurements in the presence of field inhomogeneities.

## Methods

**Figure 1:**
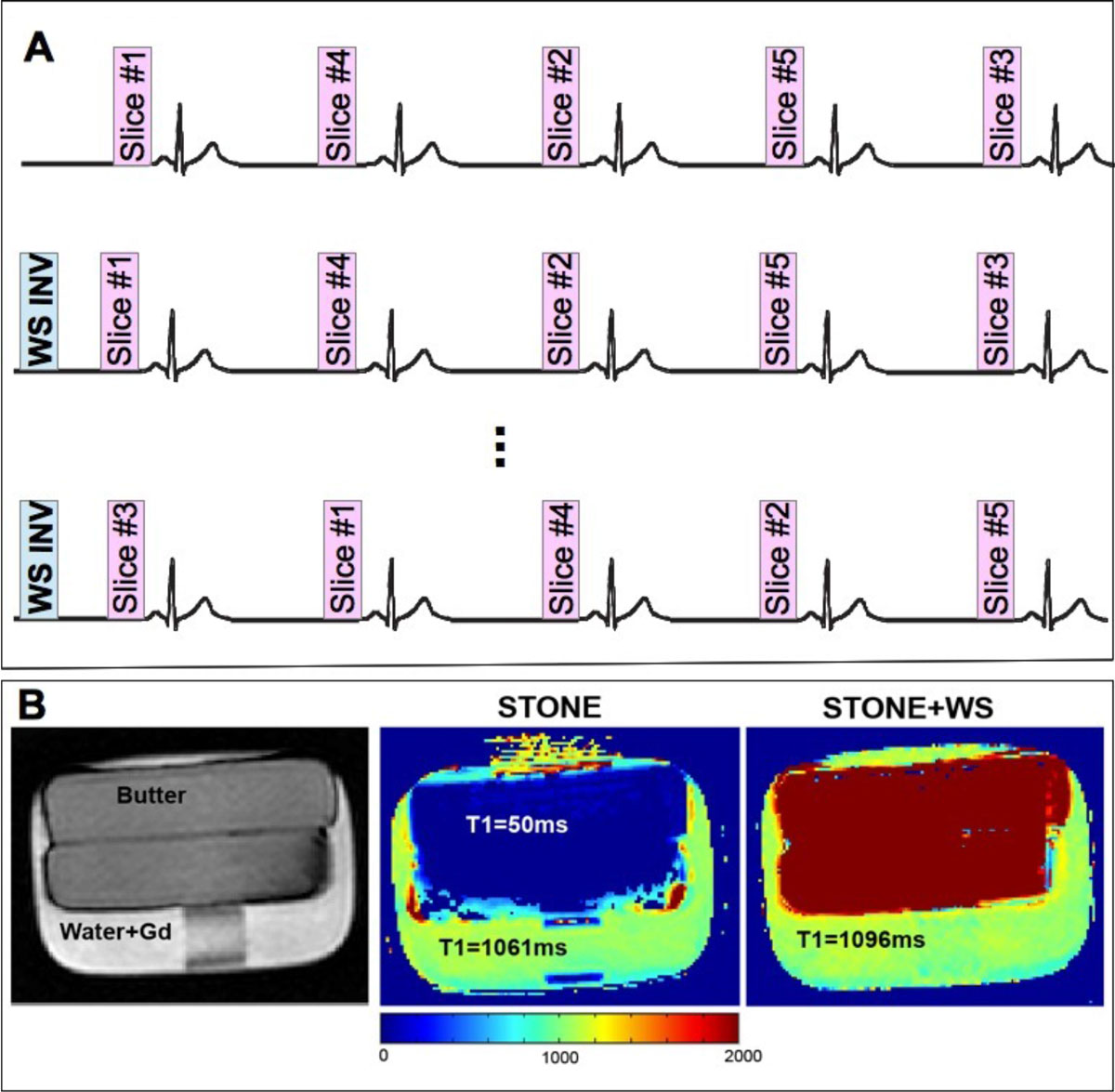
**A) Schematic of the STONE sequence with water selective RF pulse**. After a water selective inversion pulse 5 slices are acquired (TI_1_: first Inversion time). Subsequently, the slice order is changed to sampling of all 5 heartbeats for each slice. The same experiment is repeated with TI_2_. 11 images acquired for each slice. WS INV: water selective inversion pulse. **B**) The phantom consists of butter in Gd-doped water. The T_1_ of the water with Gd is 1061 ms and butter is 50 ms.

We used the recently proposed STONE T_1_ mapping sequence [5] using a gradient echo imaging readout. A water selective inversion pulse (f_0_ = 42.5 MHz, duration = 7 ms) was used instead of the adiabatic inversion pulse to only invert the water signal, and therefore, maintains signal from fat. This combination (WS-STONE) allows to perform T_1_ mapping of 5 slices and to evaluate the T1 relaxation time of the myocardium without the effect of the fat signal in vivo. The schematic of the proposed sequence is shown in Fig 1A. Numerical simulations were performed to study the effect of B_0_ and B_1_ inhomogeneities on the efficiency of the inversion pulse and thus on the T_1_ measurements of the sequence. The feasibility of the proposed sequence has been reported previously in a T_1_ mapping phantom and healthy volunteers [2]. Phantom imaging was performed using butter immerged in a bottle of Gd-doped water to evaluate the efficiency of the sequence in voxels containing both fat and water. In vivo images were also acquired in 9 healthy subjects using a 1.5T Philips Achieva scanner equipped with a 32-element cardiac coil. Scans were performed using free-breathing multi-slice T_1_ mapping sequence with a respiratory navigator. The imaging parameters included TR/TE = 4.5/2.2 ms, flip angle = 10°, FOV = 300 × 300 mm^2^, voxel size = 2 × 2 mm^2^, slice thickness = 8 mm and SENSE factor = 2. To generate the T_1_ map, a 3-parameter fit model was used.

## Results

**Figure 2:**
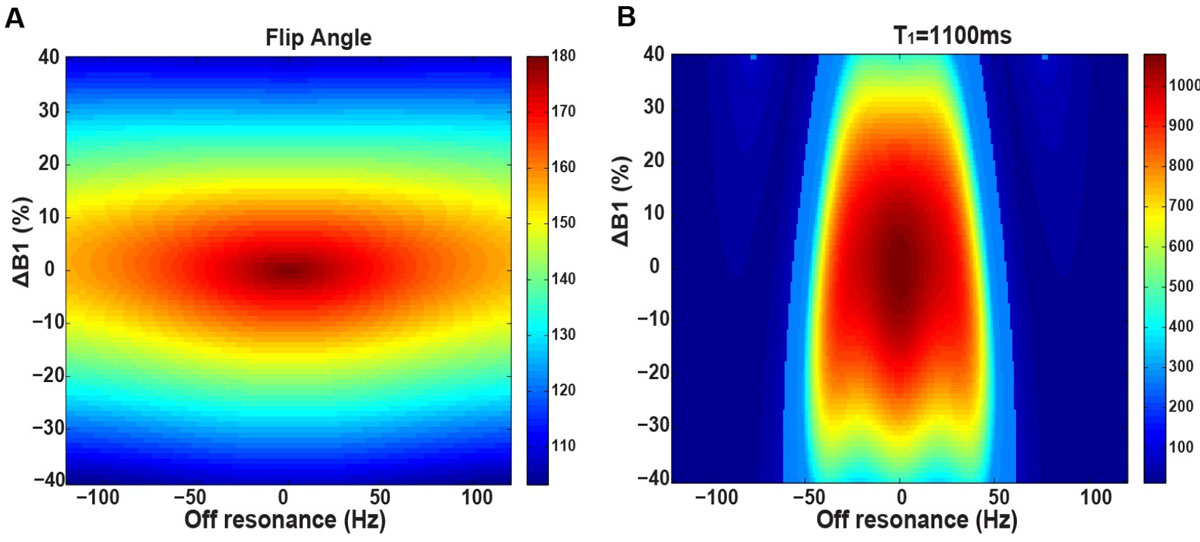
**Numerical simulation results for the effect of B**_**0**_ **and B**_**1**_ **off-resonance on A) the inversion flip angle, and B) the measured T1 value for a typical myocardial T1 value of 1100 ms**.

A representative T_1_ map of the butter phantom acquired with STONE and WS-STONE is shown in Figure 1B. The measured T_1_ of butter with the WS-STONE is infinity due to the fact that the fat signal is not disturbed during the magnetization preparation with the water selective inversion pulse. Results of numerical simulations incorporating the effect of the B_0_ and B_1_ inhomogeneity on the flip angle and measured T_1_ are shown in Fig 2, demonstrating only little changes in the flip angle for the clinically expected inhomogeneities. In vivo T1 mapping of the heart demonstrated good agreement between the two T1 mapping methods (1125 ± 27 ms for STONE vs. 1115 ± 26 ms for WS-STONE, p = 0.2).

## Conclusions

We demonstrate the feasibility of a water selective RF inversion pulse to reduce the fat impact on cardiac T_1_ measurements within the clinically expected range of field inhomogeneities.

